# Genome-Wide Identification and Characterization of the *CDPK* Family of Genes and Their Response to High-Calcium Stress in *Yinshania henryi*

**DOI:** 10.3390/genes16010109

**Published:** 2025-01-20

**Authors:** Liangfeng An, Huihui Fang, Ximin Zhang, Jing Tang, Jiyi Gong, Yin Yi, Ming Tang

**Affiliations:** 1 School of Life Sciences, Guizhou Normal University, Guiyang 550025, China; 222100100377@gznu.edu.cn (L.A.); 232100100390@gznu.edu.cn (H.F.); zhxm409@gznu.edu.cn (X.Z.); tangjing2016@gznu.edu.cn (J.T.); 201307048@gznu.edu.cn (J.G.); 2 Key Laboratory of National Forestry and Grassland Administration on Biodiversity Conservation in Karst Mountainous Areas of Southwestern China, Guizhou Normal University, Guiyang 550025, China; gzklppdr@gznu.edu.cn; 3 Engineering Research Center of Carbon Neutrality in Karst Areas, Ministry of Education, Guizhou Normal University, Guiyang 550025, China

**Keywords:** *Yinshania henryi*, *CDPK* family genes, cis-acting elements, abiotic stress, high-calcium treatment

## Abstract

**Background/Objectives:** Calcium-dependent protein kinases (CDPKs) are a crucial class of calcium-signal-sensing and -response proteins that significantly regulate abiotic stress. *Yinshania henryi* is a member of the Brassicaceae family that primarily grows in the karst regions of southwestern China, with a notable tolerance to high-calcium soils. Currently, the function of the *CDPK* family of genes in *Y. henryi* has yet to be explored. **Methods:** This study employed a comprehensive approach starting with bioinformatic methods to analyze the whole-genome sequencing data of *Y. henryi* and identified *YhCDPK* genes by combining phylogenetic characteristics and protein domain analysis. **Results:** It then delved into the physicochemical properties, gene structure, chromosomal localization, phylogenetic tree, and promoter cis-acting elements of these *YhCDPK* genes. Subsequently, RNA-seq data and qRT-PCR analysis were utilized to understand the expression patterns of *YhCDPK* genes. Twenty-eight *YhCDPK* genes were found to be unevenly distributed across six chromosomes; these can be classified into four subfamilies, with many cis-acting elements in their promoter regions involved in plant growth and stress responses. Furthermore, the differential expression patterns of *YhCDPK* genes under different concentrations of calcium treatments were investigated using RNA-seq data and qRT-PCR analysis. **Conclusions:** These results are a critical first step in understanding the functions of *YhCDPK* genes, and they lay a foundation for further elucidating the adaptability and response mechanism of *YhCDPK* genes in *Y. henryi* to the karst environment.

## 1. Introduction

Calcium is essential for plant growth and serves as an important secondary messenger, mediating various signaling pathways. It plays a crucial role in plant development and responses to biotic and abiotic stress [1]. When plant cells detect external stimuli, the concentration of free Ca^2+^ in the cytoplasm changes, activating calcium-binding proteins or calcium-signal sensors, which triggers downstream events [2]. Three families of calcium-sensor proteins exist in plants: calmodulin/calmodulin-like proteins (CaM/CMLs), calcium-dependent protein kinases (CDPKs), and calcineurin B-like proteins with their interacting protein kinases (CBL-CIPKs) [3]. These proteins collectively transmit and amplify calcium signals, promoting the production of response proteins that regulate plant growth, immune responses, and stress reactions [4,5,6].

Among these, CDPKs are serine/threonine protein kinases that directly convert Ca^2+^ signals into protein phosphorylation events [7,8]. CDPKs have four functional domains: the serine/threonine protein kinase domain (PKD), the junction domain (JD), the EF-hand motif (C–terminal), and the variable N-terminal domain (VNTD), with the latter being crucial for their function [9,10]. *CDPK* genes have been identified across various plants through whole-genome analysis, revealing significant differences in the number of *CDPK* genes among species: 34 *AtCDPK* genes in *Arabidopsis thaliana* [11], 31 *OsCDPK* genes in *Oryza sativa* [12,13], 20 *TaCDPK* genes in *Triticum aestivum*, 35 *ZmCDPK* genes in *Zea mays*, and as many as 63 *AvCDPK* genes in *Actinidia valvata* [14,15,16]. *CDPK* genes are widely distributed, with varying gene expression levels detected in multiple organs, including roots, stems, leaves, flowers, fruits, and seeds [17]. In plant development, *CDPK* genes play vital roles in responses to biotic and abiotic stress signals, regulating major processes, including pollination, growth, flowering, and senescence [18,19,20]. Environmental factors like low temperature, light, and drought, along with hormones such as gibberellins, auxins, and cytokinins, can specifically induce *CDPK* gene expression [21,22,23,24,25]. CDPK substrates include transcription factors, heat-shock proteins, protein phosphatases, and ion-channel proteins, which interact with CDPK to amplify and transmit Ca^2+^ signals to regulate gene expression, enzyme metabolism, and transmembrane transport of ions and water, thus enabling plant adaptation [26].

China’s karst landscapes, primarily in the southwestern provinces of Guizhou, Guangxi, Sichuan, and Yunnan, cover 1.3 million square kilometers. The parent rock in these areas is mainly carbonate rock, which is characterized by high concentrations of calcium, magnesium, and other elements compared with non-karst regions [27]. Plants cannot escape environmental stress and must use mechanisms to respond, tolerate, resist, or avoid adverse conditions while balancing growth and stress responses [28]. Many plants in karst areas exhibit drought tolerance, calcium tolerance, and possess thick cuticles and wax layers [29].

*Y. henryi*, a karst plant found on limestone in the Guizhou province, belongs to the Brassicaceae family and is considered a relatively primitive group in the Brassicaceae family [30]. Karst soils are notably high in calcium content, and the absorption of calcium ions by plants is proportional to the amount of exchangeable Ca^2+^ in the soil solution. Previous research showed that the karst dry–hot valley has a total soil calcium content of 13.00 ± 3.28 g/kg, and the proportion of exchangeable calcium to total calcium is 50.31% [31]. These calcium-rich soils can lead to excessive calcium absorption by plant cells. However, functional roles of the CDPK family in *Y. henryi* are still poorly understood. This study employed bioinformatic methods to identify genes of the CDPK family in *Y. henryi*. We systematically analyzed the gene structure, chromosomal distribution, conserved motifs, promoter cis-acting elements, and genetic regulatory networks of this gene family. Additionally, we investigated the responses of different *YhCDPK* gene members to varying external calcium levels, providing valuable insights for future research on the response mechanisms of *YhCDPK* genes in *Y. henryi* within the karst environment.

## 2. Results

### 2.1. Identification, Chromosomal Location, and Gene Duplication Analysis of YhCDPK Genes

We used the AtCDPK protein sequences from *A. thaliana* as query sequences and compared these against all protein sequences of *Y. henryi*, resulting in the identification of 29 candidate YhCDPK protein sequences. The details of 29 sequences are listed in Supplementary Table S1. All sequences were further compared with reference sequences from the SwissProt database obtained from UNIPROT. By integrating data from the PFAM, PROSITE PROFILES, and SMART databases. One sequence was found to contain only the STK-C domain, while lacking the EF-hand domains. Ultimately, 28 *YhCDPK* genes were identified from the whole-genome sequencing data of *Y. henryi*, which were sequentially named YhCDPK1–28.

**Figure 1:**
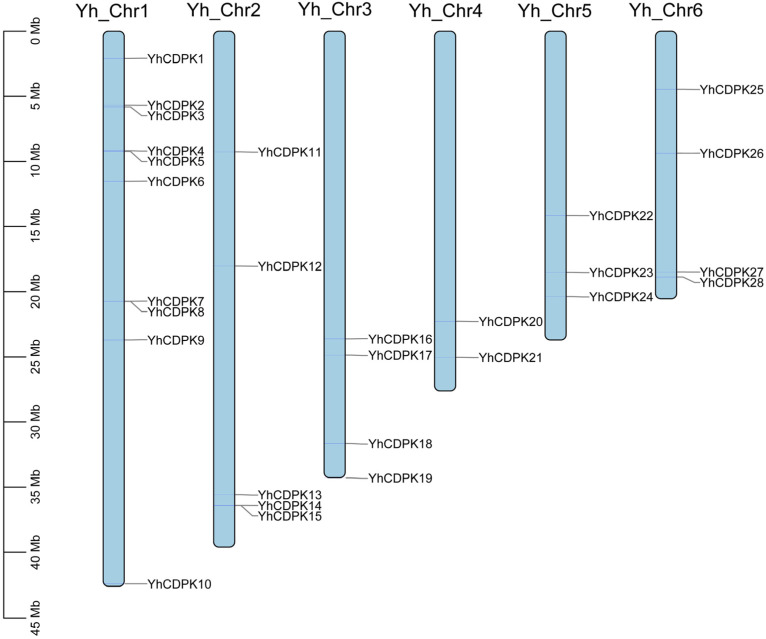
Chromosomal location distribution of the 28 *YhCDPK* genes in the *Y. henryi* genome. Chromosome numbers are listed above, and the chromosome sizes are indicated on the left of the figure. The length of each chromosome was estimated in mega bases (Mb). Detailed chromosomal location information is listed in Supplementary Table S2.

Next, as shown in Figure 1, the chromosomal location distribution of the *YhCDPK* genes were analyzed by using the Gene Location Visualize tool from the GTF/GFF plugin. *YhCDPK1–10* are located on chromosome 1 (Chr1), *YhCDPK11–15* on chromosome 2 (Chr2), *YhCDPK16–19* on chromosome 3 (Chr3), *YhCDPK20–21* on chromosome 4 (Chr4), *YhCDPK22–24* on chromosome 5 (Chr5), and *YhCDPK25–28* on chromosome 6 (Chr6).

**Figure 2:**
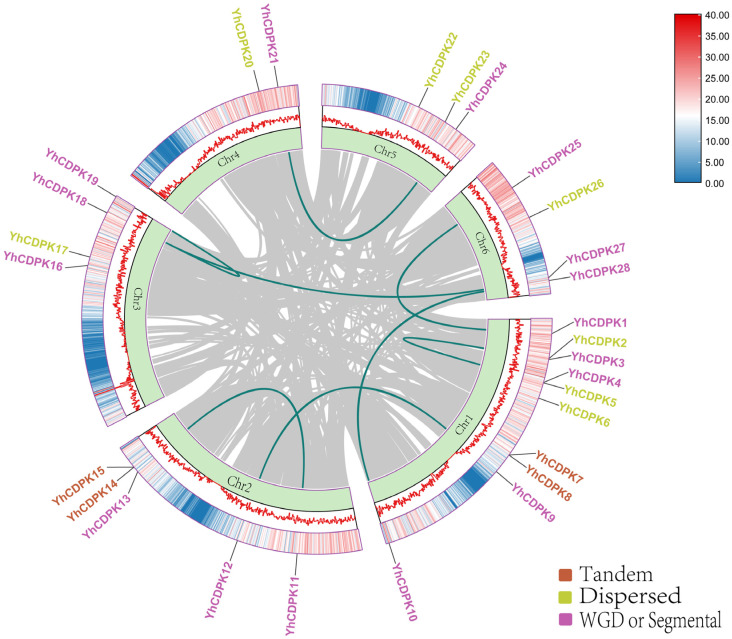
Whole-genome duplication distribution of the *YhCDPK* genes in *Y. henryi*. The color scale and lines represent the gene density on the chromosomes. The *YhCDPK* gene names are listed on the chromosomes. The gray lines in the background represent synteny blocks in the capsicum genome, while the green lines between chromosomes indicate segmental duplicated gene pairs. Detailed duplication information on the *YhCDPK* genes is listed in Supplementary Table S3.

To investigate the expansion of the *YhCDPK* genes, we utilized TBtools-II software (v2.136) to construct a duplication relationship map of the 28 *YhCDPK* genes on the chromosomes of *Y. henryi*. As shown in Figure 2, among the 28 *YhCDPK* genes, 8 are scattered duplicates distributed across Chr1 to Chr6, while 16 exhibit segmental or fragment duplications across all chromosomes. Additionally, four *YhCDPK* genes (*YhCDPK*7, *YhCDPK*8, *YhCDPK*14, and *YhCDPK*15) are tandemly duplicated on Chr1 and Chr2. These findings suggest that some *YhCDPK* genes may have originated from gene duplication events, with segmental duplication being the primary mechanism driving the evolution of the *YhCDPK* genes.

### 2.2. Physicochemical Properties of YhCDPK Proteins

The physicochemical properties of these YhCDPK proteins were analyzed based on their amino acid sequences, as summarized in Table 1. The amino acid lengths of the 28 YhCDPK proteins range from 441 to 624 residues; YhCDPK25 has the longest sequence at 624 residues, while YhCDPK6 is the shortest at 441 residues. The molecular weights of these genes vary from 50.0 kDa to 69.9 kDa. Notably, YhCDPK28 exhibited a high isoelectric point (pI) of 9.38, whereas the pI values of the other 27 CDPK proteins ranged between 5 and 7. The grand average hydropathy (GRAVY) values for all 28 YhCDPK proteins were less than 0, indicating that they are hydrophilic proteins.

All 28 YhCDPK proteins contain a serine/threonine protein kinase domain and four EF-hand domains. Compared with the 34 AtCDPK proteins in *A. thaliana*, the YhCDPK proteins display high similarity in multiple sequence alignment results. However, variable conserved motifs revealed that YhCDPK6 and YhCDPK23 exhibit structural deletions, which may be linked to their functional differences (Supplementary Figure S1).

### 2.3. Phylogenetic Analysis of YhCDPK Genes

**Figure 3:**
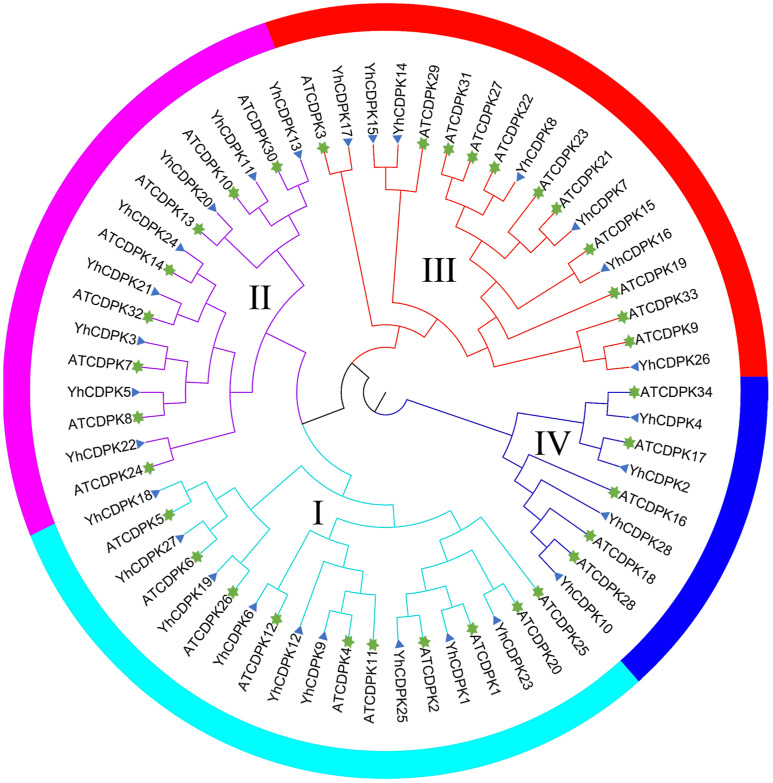
Phylogenetic analysis of the CDPKs in *Arabidopsis thaliana* and *Y. henryi*. A total of 62 CDPKs were classified into four distinct groups (Groups I to IV) based on their protein sequence structural characteristics. The different colors of the circles—green, purple, red, and blue—represent Groups I–IV, respectively. The blue triangles and green hexagonal stars represent AtCPKs in Arabidopsis and YhCPKs in *Y. henryi*, respectively.

To investigate the evolutionary relationships of CDPK proteins in different plant species, a phylogenetic tree using the Maximum Likelihood (ML) method was constructed for 62 CDPKs, including 28 YhCDPKs and 34 AtCDPKs (Figure 3). Based on the structural characteristics of protein sequences and the previous classification of CDPK proteins in *Arabidopsis*, the 28 YhCDPKs were clustered into four distinct groups, designated as Groups I to IV [11]. Group I contains 7 *YhCDPK* genes and 11 *AtCDPK* genes; Group II has the most members, comprising 8 *YhCDPK* genes and 8 *AtCDPK* genes; Group III includes 9 *YhCDPK* genes and 10 *AtCDPK* genes; while Group IV has the fewest members, with 4 *YhCDPK* genes and 5 *AtCDPK* genes. These results indicate a high degree of consistency in the species distribution of *CDPK* genes between *Y. henryi* and *A. thaliana*.

### 2.4. Conserved Motif and Exon–Intron Structure Analysis of YhCDPKs

**Figure 4:**
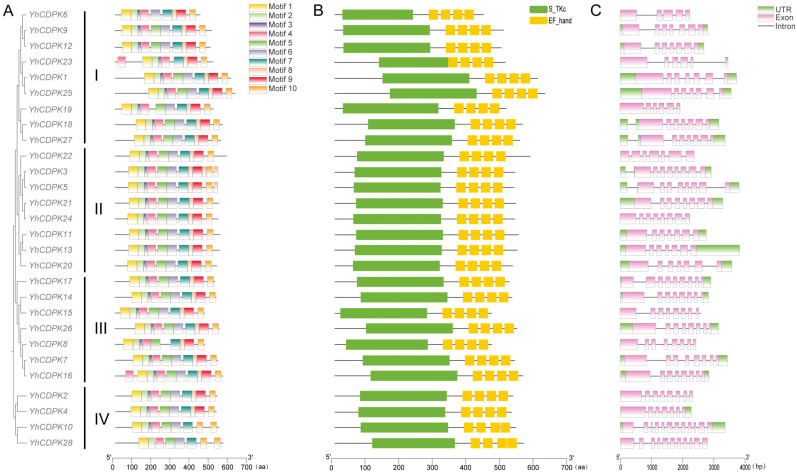
Conserved motifs and structure of the genes encoding YhCDPKs. (**A**) Motif composition and distribution of the 28 YhCDPK proteins based on their phylogenetic relationship. The branch lengths in the phylogenetic tree represent the similarity between sequences. The Roman numerals (I, II, III, and IV) represent the four distinct groups of the 28 YhCDPK proteins based on their phylogenetic analysis. The ten motifs are indicated by different colors. The lengths of the boxes and lines are proportional to the protein lengths. (**B**) Structural domain analysis of the The S_TKc domain and EF-hand domain are indicated by the green and yellow boxes, respectively. The lengths of the boxes and lines are proportional to the protein lengths. (**C**) Structure analysis of YhCDPK gene sequences. The UTR and exons are indicated by green and purple boxes, respectively, and introns are shown by black lines. The lengths of the boxes and lines are proportional to the gene lengths.

To further explore the protein structure and functional diversity of YhCDPKs, we employed the MEME online tool to identify conserved motifs among the 28 YhCDPK proteins. A total of 10 motifs were identified (Figure 4A). Overall, the higher the homology, the more substantial the similarity of the gene motif arrangement. There are notable differences in the motifs present in different YhCDPK proteins. Specifically, only motifs 2, 3, 4, 7, 8, and 10 are found in all 28 YhCDPK proteins. The motif order of YhCDPK16 and YhCDPK23 differ from the others, and they contain two copies of motif 4, one of which precedes motif 1. The motif information is available in Supplementary Table S4. Overall, the structure of YhCDPK proteins is relatively conserved, and all of the YhCDPKs contain one S_TKc domain and four EF-hand domains (Figure 4B). The gene structure varies among the 28 *YhCDPK* genes. The number of exons in the coding sequence (CDS) range from five (*YhCDPK6*) to 12 (*YhCDPK10*). However, there are considerable differences in the number of exons between Group I (5–7 exons), Group II (7–8 exons), Group III (8–9 exons) and Group IV (7–12 exons). The smallest group (IV) consists of four *YhCDPK* genes (*YhCDPK2/4/10/28*), and it presents the most significant number of exons and introns compared with the other three groups (Figure 4C). The variations among the groups suggest potential functional differences, while the conserved motifs within the groups may indicate close evolutionary relationships.

### 2.5. Synteny Analysis of CDPK Genes Between Y. henryi and Other Species

**Figure 5:**
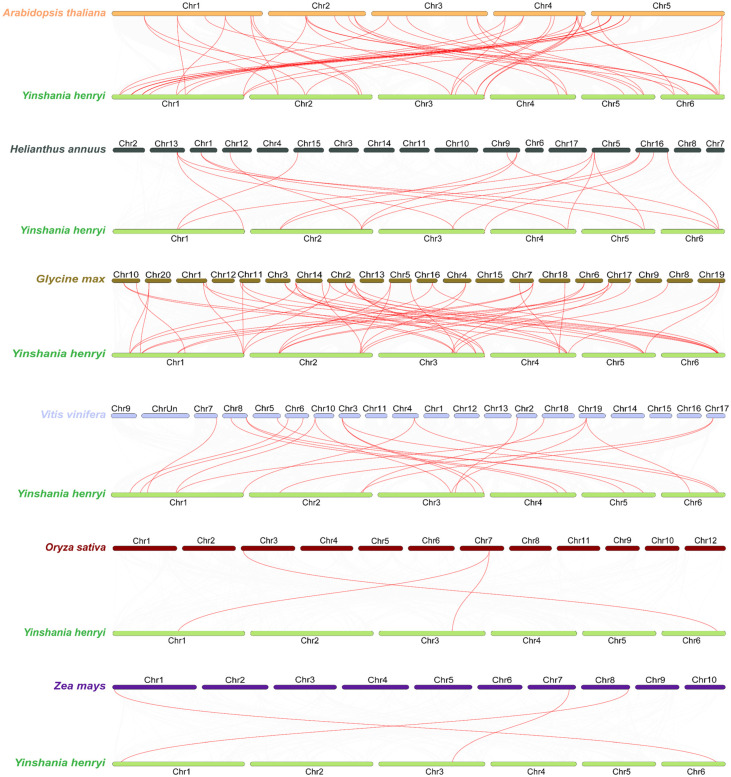
Syntenic analysis of *CDPK* genes between *Y. henryi* and other six species (*A. thaliana*, *G. max*, *H. annuus*, *V. vinifera*, *O. sativa*, and *Z. mays*). Red lines indicate collinearity relationships between the *CDPK* genes from *Y. henryi* and the other six species. The horizontal columns indicate chromosomes, with their corresponding chromosome numbers shown below the diagram.

To further elucidate the phylogenetic mechanisms of *YhCDPK* genes, a comparative map was constructed of the *YhCDPK* genes and *CDPK* genes from six other species: four dicotyledons (*A. thaliana*, soybean (*Glycine max*), sunflower (*Helianthus annuus*), and grape (*Vitis vinifera*)), and two monocotyledons (rice (*O. sativa*) and maize (*Z*. *mays*)). As shown in Figure 5, the collinearity analysis revealed that 26 *YhCDPK* genes were homologous to *AtCDPK* genes in *A. thaliana*, 19 *YhCDPK* genes were homologous to *VvCDPK* genes in *V. vinifera*, 17 *YhCDPK* genes showed homology to *GmCDPK* genes in *G. max*, and 12 *YhCDPK* genes were homologous to those in *H. annuus*. In contrast, only three *YhCDPK* genes were homologous to *CDPK* genes in both *O. sativa* and *Z. mays*. These results indicate that the collinearity and homology of *CDPK* genes between *Y. henryi* and dicotyledons are significantly greater than those with monocotyledons, suggesting that these orthologous genes likely formed after the divergence between dicotyledons and monocotyledons.

### 2.6. Analysis of Cis-Acting Elements of YhCDPK Genes

Cis-acting elements in the promoter regions upstream of gene-coding sequences play a crucial role in regulating gene transcription levels. During plant growth and development, as well as in response to external stresses, these elements interact with transcription factors to modulate gene expression. To investigate the role of *YhCDPK* genes in response to external stresses, we extracted the 2000 bp upstream sequences of the promoter regions of the 28 *YhCDPK* genes and analyzed them for cis-acting elements using the PlantCARE online tool.

**Figure 6:**
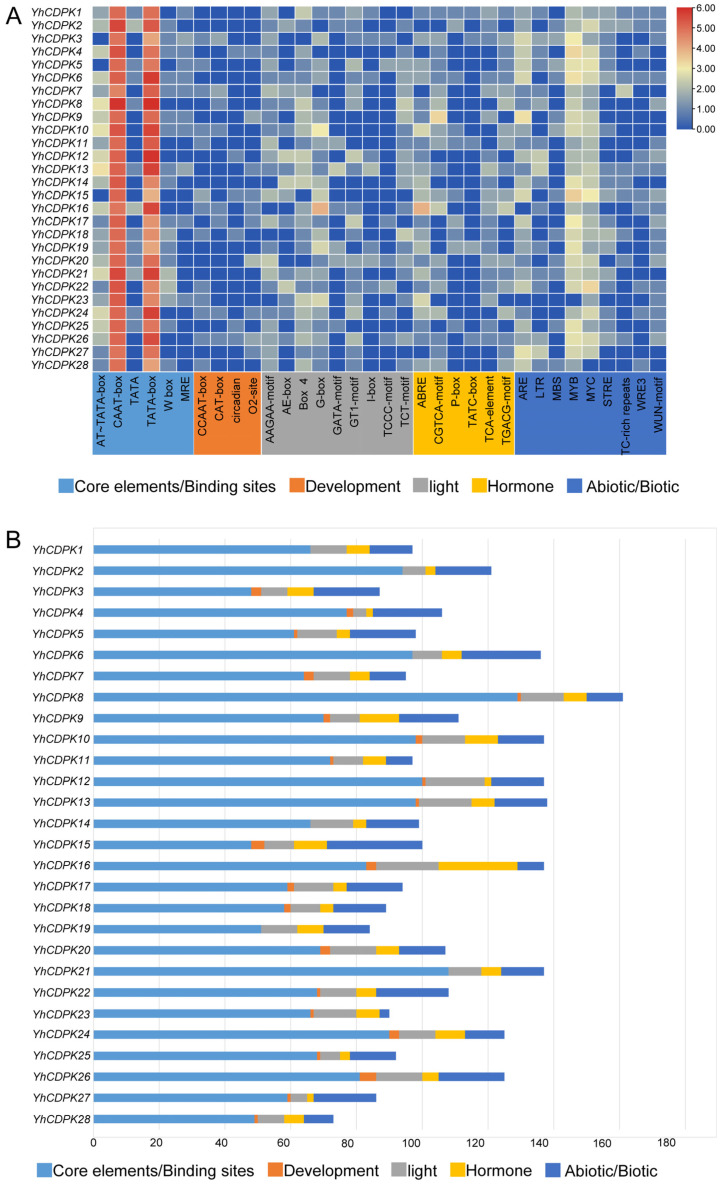
Cis-acting elements in the promoters of 28 *YhCDPK* genes. (**A**) The 34 cis-acting elements of the five types of motifs identified in the 28 *YhCDPK* genes within the 2 kb *YhCDPK* promoter regions are highlighted and represented. The color scale represents the number of cis-acting regulatory elements; (**B**) The total number of the five types of motifs of the 28 *YhCDPK* genes. Detailed information of all the cis-acting elements are listed in Supplementary Table S5.

The results, presented in Figure 6, indicate that the promoter regions of all 28 *YhCDPK* genes contain numerous core promoter elements, including the TATA-box, CAAT-box, AAGAA-motif, and TATC-box (Figure 6A,B). Additionally, we identified several hormone-responsive elements, such as ABRE, AuxRR-core, GARE-motif, TGA-box, TATC-box, TCA-element, and TGACG-motif, with the ABA-responsive element ABRE being the most abundant. Each *YhCDPK* gene contains two or more hormone-responsive elements, suggesting that these genes may be involved in responses to various plant hormones. Furthermore, a significant number of light-responsive elements (Box4, AAGAA motif, TCT motif, TCCC motif, and G-box) were found in the promoter regions of *YhCDPK* genes, with the G-box being the most prevalent. Most *YhCDPK* genes also contained anaerobic responsive elements (ARE) and drought-responsive elements (MBS, MYB, and MYC). Low-temperature-responsive elements (LTR) were identified in 17 *YhCDPK* genes, while stress-responsive elements (STREs) were present in 18 *YhCDPK* genes. Additionally, a small number of defense and damage-responsive elements (WUN, WRE3) were detected in *YhCDPK* genes.

These findings suggest that *YhCDPK* genes may play a significant role in regulating hormone signaling pathways, thereby controlling growth and development and enhancing adaptability to biotic and abiotic stresses.

### 2.7. YhCDPK Gene Expression Analysis After High-Ca^2+^ Stress

**Figure 7:**
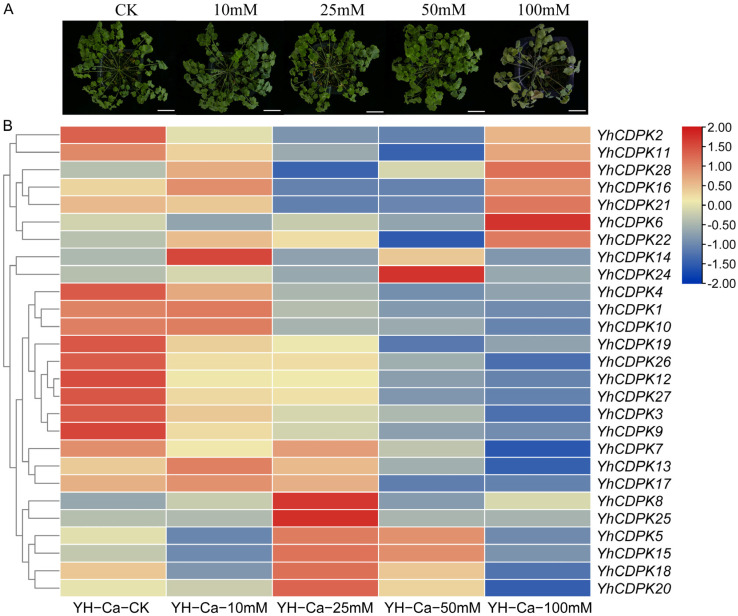
Expression pattern of *YhCDPK* genes under CaCl_2_ treatment by RNA-seq sequencing. (**A**) Phenotypes of *Y. henryi* seedlings following the different treatments; bar = 1 cm. (**B**) RNA−seq data determined the relative expression levels. CK (control), YH−Ca−10 mM (10 mM CaCl_2_), YH−Ca−25 mM (25 mM CaCl_2_), YH−Ca−50 mM (50 mM CaCl_2_), and YH−Ca−100 mM (100 mM CaCl_2_) represent different concentration treatments. The transcript abundance level was normalized and hierarchically clustered using log 2 (FPKM + 1) comparison among the genes for the different treatments. The expression value is presented on the color scale, with red representing high expression and blue representing low expression.

Next, we examined the expression of all of the *YhCDPK* genes. Two-month-old *Y. henryi* seedling plants from multiple generations of self-crossing were grown in a thermostatic greenhouse and treated with different concentrations of CaCl_2_ (CK (control; cultivated with 1/2 MS nutrient solution), 10 mM, 25 mM, 50 mM, and 100 mM) for 14 days, and leaf tissues were collected for RNA-seq sequencing analysis. With the increase in calcium concentration, especially following high-concentration treatment (100 mM), the leaves gradually turned yellow and withered. At a medium concentration (50 mM), there was no significant difference in color and height compared with CK treatment (Figure 7). RNA from 15 plant samples were extracted and sequenced, producing 108 GB of data via Illumina Hiseq3000 paired-end sequencing. In total, 20.90–58.23 million clean-read pairs were obtained from each sample (Table 2). All samples had Q30 values greater than 87.3%, and the GC content ranged from 46.3% to 48.1% (Table 2).

The number of differentially expressed genes (DEGs) increased with the increase in CaCl_2_ treatment concentration and showed a significant increase following high-concentration treatment (100 mM). As shown in Supplementary Table S6, compared with CK and the 10 mM treatment, only 11 DEGs were obtained, of which 7 were up-regulated and 4 were down-regulated. Compared with CK and the 25 mM treatment, 181 DEGs were up-regulated, and 71 DEGs were down-regulated out of a total of 252 DEGs. Compared with CK and the 50 mM treatment, 125 genes were up-regulated, and 60 genes were down-regulated out of a total of 185 DEGs. Compared with CK and the 100 mM treatment, 2437 were up-regulated, and 2299 were down-regulated out of a total of 4736 DEGs (Supplementary Figure S2).

In order to understand the function of these DEGs and the metabolic pathways involved, GO (gene ontology) and KEGG (Kyoto Encyclopedia of Genes and Genomes) enrichment analyses were performed. GO analysis of DEGs of CK-10 mM were mainly enriched in the following biological processes: photosynthetic electron transport chain, chlorophyll binding, electron transfer activity, photosynthesis, and the generation of precursor metabolites and energy, respectively (Supplementary Figure S3A). DEGs of CK-25 mM were mainly enriched in the following biological processes: cell walls, plant-type cell walls, cell-wall biogenesis, and cell-wall organization, respectively (Supplementary Figure S3B). DEGs of CK-50 mM were mainly enriched in the following biological processes: chlorophyll binding, cell wall, photosynthetic electron transport chain, oxidoreductase activity, and cuticle development, respectively (Supplementary Figure S3C). DEGs of CK-50 mM were mainly enriched in the following biological processes: response to oxygen-containing compounds, response to water deprivation, catalytic activity, vacuoles, and oxidoreductase activity, respectively (Supplementary Figure S3D).

The KEGG analysis of DEGs of CK-10 mM showed that they were mainly enriched in the following pathways: photosynthesis (Supplementary Figure S4A). DEGs of CK-25 mM were mainly enriched in the following pathways: pentose phosphate pathway; glycine, serine, and threonine metabolism; cutin, suberine, and wax biosynthesis; tyrosine metabolism; and phenylalanine metabolism, respectively (Supplementary Figure S4B). DEGs of CK-50 mM were mainly enriched in the following pathways: photosynthesis; cutin, suberine, and wax biosynthesis; phenylpropanoid biosynthesis; starch and sucrose metabolism; glutathione metabolism; and carotenoid biosynthesis, respectively (Supplementary Figure S4C). DEGs of CK-100 mM were mainly enriched in the following pathways: photosynthetic antenna proteins, MAPK signaling pathway-plant, phenylalanine metabolism, glutathione metabolism, plant hormone signal transduction, and carotenoid biosynthesis, respectively (Supplementary Figure S4D).

The GO enrichment results were partially similar to the KEGG pathway enrichment results. Photosynthetic pathways were generally enriched in the GO enrichment results and also appeared in the KEGG enrichment results for CK-10 mM, CK-50 mM, and CK-100 mM, which suggested that the effect of calcium treatment on the photosynthesis of plants is universal. In addition, the glutathione metabolic pathway, which has the function of clearing ROS to avoid oxidative damage to cells, was enriched in the KEGG enrichment results of CK-50 mM and CK-100 mM, similar to the results of GO enrichment, indicating that oxidative damage caused by calcium treatment mainly occurred following high-concentration treatment.

As shown in Figure 7, the 28 *YhCDPK* genes exhibited distinct expression patterns in response to calcium treatment. Among the 28 *YhCDPK* genes, the RNA-seq data did not provide expression information for the *YhCDPK23* gene, suggesting that it may only be expressed during early developmental stages, such as seed germination. The expression of most genes showed a tendency for down-regulation with the increase in calcium concentration. The expression of seven *YhCDPK* genes (*YhCDPK2/6/11/16/21/22/28*) showed a tendency of first falling and then rising, with the highest period of expression occurring in the high-concentration 100 mM calcium treatment. Four *YhCDPK* genes (*YhCDPK7/13/17/26*) showed a significant tendency for down-regulation with the increase in calcium concentration. Six *YhCDPK* genes (*YhCDPK5/8/15/18/20/25*) showed a tendency of first rising and then falling. Interestingly, *YhCDPK14* displayed different expression patterns. We analyzed these *YhCDPK* proteins’ structure, the total number of cis-acting elements, and the number of cis-acting elements of abiotic/biotic stress; the results showed that there were no significant differences between these *YhCDPK* genes, which suggest that there are fine and complex mechanisms for activating and regulating *YhCDPK* genes.

**Figure 8:**
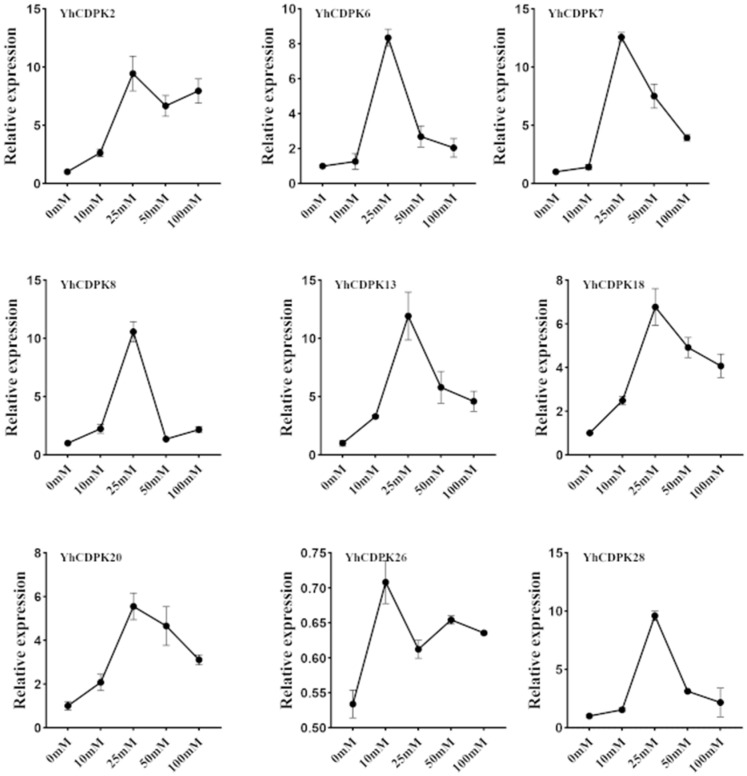
Expression analysis of *YhCDPK* genes with real-time q-PCR following CaCl_2_ treatment. The standard errors from three biological and three technical replications are presented as error bars.

To confirm the expression patterns of the *YhCDPK* genes, we selected 10 of the *YhCDPK* genes (Group I: *YhCDPK6, YhCDPK23*, and *YhCDPK18*; Group II: *YhCDPK13* and *YhCDPK20*; Group III: *YhCDPK7*, *YhCDPK8*, and *YhCDPK26*; Group IV: *YhCDPK2* and *YhCDPK28*) and performed quantitative real-time PCR (qRT-PCR) on these. As with RNA-seq sequencing, two-month-old plants were treated with different concentrations of CaCl_2_ (CK, 10 mM, 25 mM, 50 mM, and 100 mM) for 14 days, and leaf tissues were collected for RNA isolation, reverse transcription, and amplification. As shown in Figure 8, there was still no amplification signal for *YhCDPK23*, which suggests that *YhCDPK23* may not be expressed in the leaf tissues. Overall, the results of the qRT-PCR analysis were consistent with those of the RNA-seq sequencing. Expression of all the *YhCDPK* genes peaked with the 25 mM treatment, was downregulated with the 50 mM treatment, and showed a different pattern with the 100 mM treatment.

## 3. Discussion

Calcium (Ca^2+^), a ubiquitous second messenger in plant signaling systems, plays a crucial role in plant growth, development, and responses to biotic and abiotic stress [32,33]. In plant cells, calcium exists in three forms: free, bound, and stored, with total calcium content ranging from 0.1 to 10 mmol/L. These forms affect various cellular functions through concentration changes over time and space [34]. The calcium content in plants varies significantly, ranging from 0.1% to 5%, with higher concentrations found in cell walls compared with the cytoplasm. This low cytoplasmic Ca^2+^ is vital for plant growth and development [35]. Key functions of calcium ions include stabilizing cell membrane and wall structures and regulating the osmotic balance of anions and cations within vacuoles [36]. Environmental factors such as temperature, light, salt, and osmotic stress can trigger changes in calcium-ion levels. Specific calcium receptors detect these changes, subsequently regulating gene expression through a cascade of reactions [37].

When the cells are stimulated, the increase in the concentration of calcium ions in the cytoplasm or other cellular regions forms the calcium signal, which takes on a unique shape, mainly determined by the amplitude, duration, storage location of calcium ions, and distribution in different locations within the cell [38]. Calcium signals must be interpreted by calcium sensors and translated into specific biochemical reactions. CDPK is a class of serine/threonine protein kinases widely found in plants, which is a crucial component in the Ca^2+^ signaling cascade, playing significant roles in plant growth and development and inducing protective responses to environmental stress [4,5,6]. In this study, we identified 28 *YhCDPK* genes from the whole-genome sequencing data of *Y. henryi*. Phylogenetic analysis showed that like in many plants [12,16,39,40,41], *YhCDPK* genes can be classified into four groups, indicating a common feature between species. Among them, Group IV consists of four *YhCDPK* genes (*YhCDPK*2, *4*, *10*, *28*), consistent with the small number of CDPK genes in other plants from Group IV. There is only one *CDPK* gene in Group IV of Cili (*Rosa roxburghii* Tratt.), whereas there are two *CDPK* genes in Group IV of pecan (*Carya illinoinensis*) and Chinese hickory (*Carya cathayensis*) [42,43]. Previous research reported that *CDPK* genes have a single origin and can be dated back to green algae before plants colonized the land [4]. The sequence of *CDPK* gene evolution can be analyzed by the distribution of introns and exons. Group IV was the earliest to expand from the evolutionary branch, which has the most extended evolutionary history, leading to a complex intron–exon organization. In this study, the number of exons and introns of *YhCDPK10* and *YhCDPK28* in Group IV is significantly more than that of other groups, which is consistent with the results of previous studies [42,43,44]. These genes are presumed to be the earliest *YhCDPK* genes in evolution.

Previous studies have reported the essential roles of *AtCDPK17* and *AtCDPK34* in determining pollen suitability and in the enhancement of pollen tube tip growth through the transmission of Ca^2+^ signals, *AtCDPK16* regulates hypoxia-induced ROS production by phosphorylating the NADPH oxidase RBOHD [45,46]. In this study, YhCDPK2 and AtCDPK17, YhCDPK4 and AtCDPK34, and YhCDPK28 and AtCDPK16 also belong to the same branch, suggesting that they may have similar functions. Previous studies have found that the expression of the *ShCDPK* family of genes changes significantly under cold and drought stress [44]. Whether the *YhCDPK* family is involved in the regulation of plants resistance to biotic and abiotic stress is unknown. Therefore, in this study, the cis-acting elements of the promoter of *YhCDPK* genes were analyzed, and it was found that *YhCDPK* genes contained hormone-responsive elements, light-responsive elements, anaerobic responsive elements, drought-responsive elements, low-temperature-responsive elements, and stress-responsive elements, suggesting that *YhCDPK* genes may respond to stress.

Previous studies have shown that CDPKs can sense fluctuations in Ca^2+^ concentration via their EF-hand structures, release self-inhibition, activate kinase domains, and then transmit information to regulate physiological changes in plants, and they participate widely in plant growth and development and morphogenesis [47,48]. Depending on the number of EF-hand motifs and changes to their amino acid sequence, the affinity of CDPKs to Ca^2+^ may vary [49]. Although all the CDPKs discovered contain Ca^2+^ binding EF-hand domains, not every CDPK is fully regulated by Ca^2+^. Previous studies have concluded that the kinase activity of at least six *AtCDPK* genes (*AtCDPK7/8/10/13/25/30*) is basically not regulated by Ca^2+^, and studies have found that they have high kinase activity in the absence of Ca^2+^ [50]. In this study, the number of EF-hand domains contained in YhCDPK proteins was similar by domain analysis. We further analyzed the response of *YhCDPK* genes to different concentrations of calcium-ion stress. A concentration of 0 mM calcium treatment was used as the control, and it was found that a few *YhCDPK* genes showed an up-regulation effect after high-calcium stress, suggesting that these genes play an important role in responding to different calcium levels. The results of this study indicate that the *YhCDPK* genes can respond to high-calcium stress and calcium concentration changes and may induce calcium absorption and transport in plants to maintain normal physiological functions, but the specific mechanism remains to be studied. Further analysis shows that the expression of most *YhCDPK* genes was down-regulated with the increase in CaCl_2_ treatment concentration, suggesting that the role of calcium-ion sensors in *Y. henryi* may be based on the *CBL-CIPK* pathway or *CaM/CML* pathway. In the RNA-seq data, unlike the *YhCDPK* genes, the expression levels of the *YhCIPK* genes and *YhCML* genes were generally up-regulated with high-calcium treatment (Supplementary Figures S5 and S6), which also partially hinted at this conclusion.

Plants in karst habitats, such as *Y. henryi*, often exhibit unique adaptations due to the environmental heterogeneity of karst landscapes [51]. Research indicates that Ca^2+^ and Mg^2+^ concentrations in karst soils are higher than in non-karst soils. Plants living in karst landform areas have the characteristics of tolerance to a high-calcium environment. The concentration of Ca^2+^ in the root, stem, leaf, cell wall, vacuole, and other structural components of plants growing on karst calcareous soils is notably higher compared with those growing in non-karst areas [52]. In this study, after 14 days of high concentration (100 mM) of calcium stress treatment, the *Y. henryi* plants were still alive but showed yellowing and wilting. Currently, the calcium concentration inside the *Y. henryi* plant cells and the mechanism of tolerance to high calcium levels are not clear. Recent studies suggest that these conditions contribute to the adaptability of karst plants to elevated calcium and magnesium levels [53]. YhCDPK may play a critical role in this adaptation. However, this study only revealed the characteristics of *YhCDPK* genes and their expression characteristics. The molecular mechanism of YhCDPK in response to high-calcium stress is unclear. The work to be carried out in the future includes constructing a plant transgenic system using gene overexpression and CRISPR-Cas9 gene-editing technologies, as well as analyzing the proteins and transcription factors interacting with the *YhCDPK* gene through yeast hybrid technology to reveal the function of the *YhCDPK* gene. This study provides a theoretical foundation for future research on the YhCDPK protein family’s roles and the response mechanisms of *YhCDPK* genes in *Y. henryi* to the karst habitat.

## 4. Materials and Methods

### 4.1. Identification and Physicochemical Property Analysis of the CDPK Gene Family in Y. henryi

The whole-genome sequencing and annotation data of *Y. henryi* were obtained through accession number CRA019022 (https://ngdc.cncb.ac.cn/gsub/submit/gsa/list, accessed on 8 January 2025). AtCDPK protein sequences were retrieved from the TAIR database (https://www.arabidopsis.org/, accessed on 5 July 2024), while secondary alignment sequences were downloaded from the UniProt database (https://www.uniprot.org/, accessed on 5 July 2024). To identify *YhCDPK* gene family members in *Y. henryi*, we utilized the BLAST function of TBtools-II software (v2.136) with default parameters [54], using the 34 CDPK protein sequences of *A. thaliana* as queries against the 28 YhCDPK protein sequences. The resulting sequences were further validated against SwissProt protein sequences from UniProt, and domains were confirmed using the PFAM, PROSITE, and SMART databases via INTERPRO. Non-CDPK members were excluded, leading to the identification of the *YhCDPK* gene family. The “Protein Parameter Calc” (ProtParam-based) plugin of TBtools-II software (v2.136) was then used to analyze the physicochemical properties, including amino acid counts, molecular weights, isoelectric points, and instability indices.

### 4.2. Chromosomal Localization and Gene Duplication Analysis of YhCDPKs

The *YhCDPK* gene family density information on the chromosomes was extracted using the “Gene Density Profile” plugin of TBtools-II software (v2.136), followed by chromosomal localization analysis of the CDPK genes using the “Gene Location Visualize from GTF/GFF” plugin.

### 4.3. Characterization of YhCDPK Proteins

The protein sequences of YhCDPK1–YhCDPK28 were aligned and trimmed using the MEGA 11 software [55]. The YhCDPK19 sequence was modeled online via the SWISS-MODEL Interactive Workspace [56], with protein sequences analyzed online using ESPript 3 [57].

### 4.4. Distribution of Conserved Motifs in YhCDPK Proteins

Conserved motifs were analyzed online using MEME [58] (https://meme-suite.org/meme/, accessed on 8 July 2024), with parameters set to allow repeated motifs, a maximum of 10 motifs, and a minimum width of 50 amino acids. Results were visualized with TBtools-II software.

### 4.5. Phylogenetic Analysis of the YhCDPK Family Genes

A phylogenetic tree was constructed using the Maximum Likelihood (ML) method (Bootstrap = 1000; LG + G model) in MEGA 11, aligning YhCDPK sequences with *A. thaliana* CDPK sequences to assess evolutionary relationships.

### 4.6. Collinearity Analysis of YhCDPK Genes

Genome sequencing and annotation data for *A. thaliana*, *G. max* (soybean), *H. annuus* (sunflower), *V. vinifera* (grape), as well as the monocots *O. sativa* (rice) and *Z. mays* (maize), were obtained from Phytozome (https://phytozome-next.jgi.doe.gov/, accessed on 10 July 2024). The positional information of *YhCDPK* genes on chromosomes was extracted from genome annotation files. The One Step MCScanX-Super Fast plugin of TBtools-II software was used for the collinearity analysis and visualized using the Gene Location Visualize (Advanced) plugin of TBtools-II software, comparing *Y. henryi* with *A. thaliana*, maize, rice, grape, soybean, and sunflower.

### 4.7. Cis-Acting Element Analysis of the YhCDPK Family of Genes

To identify cis-acting elements regulating the *YhCDPK* genes, the 2000 bp sequence upstream of the transcription start site of the each *YhCDPK* gene was extracted from genome sequencing data using the Gtf/Gff3 Sequences Extract plugin of TBtools-II software (v2.136). Promoter sequences were predicted using the PlantCARE online tool (https://bioinformatics.psb.ugent.be/webtools/plantcare/html/, accessed on 15 September 2024) to identify cis-acting elements [59].

### 4.8. Plant Materials and Cultivation

The original *Y. henryi* plants were acquired from the KuanKuoShui National Nature Reserve in Zunyi City, Guizhou province, China, in 2022. A tiny pot (7 cm in diameter and 6 cm in height) was filled with nutrient soil (Pindstrup, Denmark) and seeds of *Y. henryi* plants were placed on the soil’s surface until the seeds germinated. The seedling plants were planted in a thermostatic greenhouse using nutrient soil containing 1/2 Murashige and Skoog (MS) nutrient solution (Duchefa Biochemie, Haarlem, The Netherlands) at 20 °C, 16 h/8 h (day/night).

### 4.9. RNA-Seq Sequencing of Y. henryi Plants with Different Concentrations of Ca^2+^ Treatment

Two-month-old *Y. henryi* plants were treated with 1/2 MS nutrient solution with different concentrations of calcium chloride (1/2 MS nutrient solution as control (CK), 10 mM, 25 mM, 50 mM, and 100 mM). On the 14th day after treatment, leaves were collected and preserved in liquid nitrogen, then sent to Wuhan Frasergen Sequencing Company for RNA-seq sequencing. FPKM was used to quantify the expression levels of genes, and differentially expressed genes were selected by Log2FC ≥ 1. The *YhCDPK* gene expression heatmap was displayed using the HeatMAP plug-in in TBtools-II software. The processed data can be obtained through accession number PRJNA1159502.

### 4.10. qRT-PCR Analysis of YhCDPK Genes

Plants treated in the same way as for RNA-seq sequencing were used for qRT-PCR analysis in this study. Three independent biological replicates were taken, each comprising three plants. Total RNA was extracted from the leaves using the TRIzol method (Invitrogen, Waltham, MA, USA) and used for mRNA synthesis with EasyScript^®^ One-Step gDNA Removal and cDNA Synthesis SuperMix Kits (TransGen Biotech, Waltham, Beijing, China) following the manufacturer’s protocol. qRT-PCR was conducted using the SYBR detection protocol (Biocontrast SciTech, Xiamen, China) on a 7500 Real-Time PCR system (Applied Biosystems, Foster City, CA, USA). The reaction mixture was composed of first-strand cDNA, primer mix, and SYBR Green M Mix (Biocontrast SciTech; code A4004M) to a final volume of 20 μL. The *Yhactin* gene was used as an internal control. The reaction conditions were as follows: denaturation, 95 °C for 5 min, followed by 40 cycles of 95 °C for 10 s and 60 °C for 30 s. The relative gene expression levels were quantified using the comparative CT method [60]. Specific primers for the qRT-PCR analysis are listed in Supplementary Table S7. The gene expression profiles presented in the Results section were constructed using GraphPad Prism software (v9.5.0).

## 5. Conclusions

Calcium is an essential macronutrient for plant growth, serving as a crucial second messenger that orchestrates responses to environmental stress. However, excessive calcium concentrations can harm plant cells, necessitating intricate mechanisms to maintain intracellular calcium homeostasis. *Y*. *henryi*, which thrives in karst regions, exhibits unique traits that enable it to tolerate elevated calcium levels in karst soils. This analysis of *YhCDPK* genes’ characteristics illuminates the environmental adaptability mechanisms of *Y. henryi*. Key findings include the following: (1) The identification of 28 *YhCDPK* genes through genome sequencing, which are fewer than in model organisms like *A. thaliana* and *O. sativa*. (2) The distribution of these genes across six chromosomes, with specific tandem distributions observed. (3) All identified YhCDPK proteins contain a serine/threonine protein kinase and four EF-hand domains. (4) Phylogenetic analysis of the 28 *YhCDPK* genes indicates that they can be grouped into four clusters, with motif structures similar to those of *CDPK* genes from other species. (5) Promoter analysis reveals several cis-acting elements critical for plant stress responses, emphasizing the role of the *YhCDPK* genes in stress adaptation. (6) RNA-seq data and qRT-PCR results confirm that *YhCDPK* genes exhibit distinct expression patterns under varying calcium-ion stress conditions.

## Figures and Tables

**Table 1:** Characterization of the YhCDPK proteins.

Protein Name	Number of Amino Acids	Molecular Weight	pI	Instability Index	Aliphatic Index	Grand Average of Hydropathicity
YhCDPK1	602	67,249.64	5.51	43.05	78.7	−0.44
YhCDPK2	530	58,692.7	5.74	37.97	78.42	−0.443
YhCDPK3	535	60,240.61	5.87	43.45	80.93	−0.46
YhCDPK4	525	58,273.19	5.76	40.91	78.42	−0.471
YhCDPK5	533	59,912.38	5.8	36.95	81.97	−0.422
YhCDPK6	441	50,032.17	5.41	42.37	84.26	−0.428
YhCDPK7	534	60,278.81	6.12	36.97	80.71	−0.47
YhCDPK8	466	52,628.88	5.54	35.02	82.88	−0.496
YhCDPK9	501	56,513.36	5.12	49.17	83.29	−0.36
YhCDPK10	538	60,856.17	8.99	40.48	83.03	−0.538
YhCDPK11	545	61,560.88	5.86	35.1	88.37	−0.321
YhCDPK12	495	55,914.98	5.31	45.07	82.93	−0.359
YhCDPK13	542	61,665.74	6.28	40.99	86.49	−0.426
YhCDPK14	526	59,600.81	6.07	39.09	78.78	−0.506
YhCDPK15	465	52,777.19	5.66	33.02	85.33	−0.472
YhCDPK16	559	63,319.09	5.85	39.76	81.65	−0.5
YhCDPK17	518	58,311.36	6.1	39.06	76.99	−0.52
YhCDPK18	558	62,365.73	5.38	36.36	85.65	−0.351
YhCDPK19	509	57,228.58	5.71	40.31	92.16	−0.207
YhCDPK20	528	59,336.82	6.36	41.37	85.3	−0.424
YhCDPK21	538	60,920.52	6.07	38.19	84.63	−0.485
YhCDPK22	580	65,976.58	6.57	43.41	81	−0.448
YhCDPK23	507	55,875.89	5.61	38.23	88.3	−0.264
YhCDPK24	534	60,492.57	6.98	35.41	87.98	−0.41
YhCDPK25	624	69,883.56	5.31	51.1	77.79	−0.476
YhCDPK26	541	60,410.49	6.03	38.33	77.93	−0.501
YhCDPK27	549	61,660.96	5.24	43.35	85.94	−0.351
YhCDPK28	560	63,442.26	9.38	37.09	77.36	−0.617

**Table 2:** Statistical summary for all the RNA-seq samples.

Sample Names	Clean-Read Pairs	Clean Base (bp)	GC Content (%)	Q30 (%)
YH-Ca-CK-1	21,258,380	6,377,514,000	46.7; 46.7	89.4; 87.3
YH-Ca-CK-2	35,431,915	10,629,574,500	46.6; 46.6	89.5; 89.1
YH-Ca-CK-3	29,021,207	8,706,362,100	46.8; 46.8	90.5; 90.1
YH-Ca-10-1	58,229,850	17,468,955,000	47.3; 47.3	89.3; 87.8
YH-Ca-10-2	24,503,299	7,350,989,700	48.1; 48.1	89.1; 88.6
YH-Ca-10-3	32,249,122	9,674,736,600	46.7; 46.7	89.7; 88.9
YH-Ca-25-1	20,897,225	6,269,167,500	46.5; 46.6	89.1; 87.3
YH-Ca-25-2	27,215,871	8,164,761,300	47.1; 47.1	88.5; 87.9
YH-Ca-25-3	29,353,856	8,806,156,800	46.8; 46.8	89.8; 90.3
YH-Ca-50-1	22,168,875	6,650,662,500	46.7; 46.8	89.6; 88.0
YH-Ca-50-2	18,451,277	5,535,383,100	46.8; 46.8	89.4; 88.3
YH-Ca-50-3	25,689,220	7,706,766,000	47.3; 47.4	88.0; 86.5
YH-Ca-100-1	28,663,857	8,599,157,100	46.4; 46.4	88.6; 87.7
YH-Ca-100-2	29,881,370	8,964,411,000	46.3; 46.4	89.4; 87.8
YH-Ca-100-3	24,022,241	7,206,672,300	46.9; 46.9	89.5; 87.5

## Data Availability

The whole-genome sequencing data presented in this study are openly available; the processed data can be obtained through accession number CRA019022 (https://www.cncb.ac.cn/search?dbId=&q=CRA019022). The RNA-seq sequencing data presented in this study are openly available; the processed data can be obtained through accession number PRJNA1159502 (https://dataview.ncbi.nlm.nih.gov/object/PRJNA1159502).

## Supplementary Materials

The following supporting information can be downloaded at: https://www.mdpi.com/article/10.3390/genes16010109/s1, Supplementary Figure S1: Amino acid sequence alignment of multiple YhCDPK and selected AtCDPK domains. Supplementary Figure S2: Comparison of DEGs in the different treatment groups. Supplementary Figure S3: Significantly enriched GO terms of the DEGs generated in the different treatment groups. Supplementary Figure S4: Significantly enriched KEGG pathways of the DEGs generated in the different treatment groups. Supplementary Figure S5: Expression pattern of *YhCBL* genes and *YhCIPK* genes under CaCl_2_ treatment by RNA-seq sequencing. Supplementary Figure S6: Expression pattern of *YhCAM* genes and *YhCML* genes under CaCl_2_ treatment by RNA-seq sequencing. Supplementary Table S1: The 29 candidate YhCDPK protein sequences of *Y. henryi*. Supplementary Table S2: Distribution of *YhCDPK* genes on chromosomes. Supplementary Table S3: Duplication type of the 28 *YhCDPK* genes of *Y. henryi*. Supplementary Table S4: Analysis and distribution of conserved motifs in YhCDPK proteins of *Y. henryi*. Supplementary Table S5: Analysis of promoter cis-acting elements of the 28 *YhCDPK* genes. Supplementary Table S6: Statistics on the differentially expressed gene analysis results. Supplementary Table S7: Primers used for the qRT-PCR analysis in this study.

## References

[1] Boudsocq M., Willmann M.R., McCormack M., Lee M., Shan L.B., He P., Bush J., Cheng S.H., Sheen J. (2010). Differential innate immune signalling via Ca^2+^ sensor protein kinases. Nature.

[2] Allan C., Morris R.J., Meisrimler C.N. (2022). Encoding, transmission, decoding, and specificity of calcium signals in plants. J. Exp. Bot..

[3] Wang K.L., Zhang C.H., Chen H.S., Yue Y.M., Zhang W., Zhang M.Y., Qi X.K., Fu Z.Y. (2019). Karst landscapes of China: Patterns, ecosystem processes and services. Landsc. Ecol..

[4] Hamel L.P., Sheen J., Séguin A. (2014). Ancient signals: Comparative genomics of green plant CDPKs. Trends Plant Sci..

[5] Romeis T., Herde M. (2014). From local to global: CDPKs in systemic defense signaling upon microbial and herbivore attack. Curr. Opin. Plant Biol..

[6] Simeunovic A., Mair A., Wurzinger B., Teige M. (2016). Know where your clients are: Subcellular localization and targets of calcium-dependent protein kinases. J. Exp. Bot..

[7] Poovaiah B.W., Du L.Q., Wang H.Z., Yang T.B. (2013). Recent advances in calcium/calmodulin-mediated signaling with an emphasis on plant-microbe interactions. Plant Physiol..

[8] Zhang Q.S., Li Z., Yang J., Li S.Q., Yang D.C., Zhu Y.G. (2012). A calmodulin-binding protein from rice is essential to pollen development. J. Plant Biol..

[9] Xu W.W., Huang W.C. (2017). Calcium-dependent protein kinases in phytohormone signaling pathways. Int. J. Mol. Sci..

[10] Dammann C., Ichida A., Hong B., Romanowsky S.M., Hrabak E.M., Harmon A.C., Pickard B.C., Harper J.F. (2003). Subcellular targeting of nine calcium-dependent protein kinase isoforms from *Arabidopsis*. Plant Physiol..

[11] Cheng S.H., Willmann M.R., Chen H.C., Sheen J. (2002). Calcium signaling through protein kinases. The *Arabidopsis* calcium-dependent protein kinase gene family. Plant Physiol..

[12] Asano T., Tanaka N., Yang G.X., Hayashi N., Komatsu S. (2005). Genome-wide identification of the rice calcium-dependent protein kinase and its closely related kinase gene families: Comprehensive analysis of the CDPKs gene family in rice. Plant Cell Physiol..

[13] Ray S., Agarwal P., Arora R., Kapoor S., Tyagi A.K. (2007). Expression analysis of calcium-dependent protein kinase gene family during reproductive development and abiotic stress conditions in rice (*Oryza sativa* L. ssp. indica). Mol. Genet. Genomics.

[14] Li A.L., Zhu Y.F., Tan X.M., Wang X., Wei B., Guo H.Z., Zhang Z.L., Chen X.B., Zhao G.Y., Kong X.Y. (2008). Evolutionary and functional study of the CDPK gene family in wheat (*Triticum aestivum* L.). Plant Mol. Biol..

[15] Ma P.D., Liu J.Y., Yang X.D., Ma R. (2013). Genome-wide identification of the maize calcium-dependent protein kinase gene family. Appl. Biochem. Biotechnol..

[16] Li Y.Y., Zhang H.X., Liang S., Chen X.L., Liu J.Y., Zhang Y., Wang A.X. (2022). Identification of CDPK gene family in *Solanum habrochaites* and its function analysis under stress. Int. J. Mol. Sci..

[17] Cheng H.K., Pan G.Y., Zhou N., Zhai Z.K., Yang L.Q., Zhu H.F., Cui X., Zhao P.Y., Zhang H.F., Li X.J. (2022). Calcium-dependent protein kinase 5 (CPK5) positively modulates drought tolerance through phosphorylating ABA-responsive element binding factors in Oilseed rape (*Brassica napus* L.). Plant Sci..

[18] Wu P., Wang W.L., Duan W.K., Li Y., Hou X.L. (2017). Comprehensive Analysis of the CDPK-SnRK superfamily genes in Chinese cabbage and its evolutionary implications in plants. Front. Plant Sci..

[19] Zhao L.N., Shen L.K., Zhang W.Z., Zhang W., Wang Y., Wu W.H. (2013). Ca^2+^-dependent protein kinase11 and 24 modulate the activity of the inward rectifying K^+^ channels in *Arabidopsis* pollen tubes. Plant Cell.

[20] Liu H.L., Che Z.J., Zeng X.R., Zhou X.Q., Sitoe H.M., Wang H., Yu D.Y. (2016). Genome-wide analysis of calcium-dependent protein kinases and their expression patterns in response to herbivore and wounding stresses in soybean. Funct. Integr. Genomics.

[21] Li W.G., Komatsu S. (2000). Cold stress-induced calcium-dependent protein kinase(s) in rice (*Oryza sativa* L.) seedling stem tissues. Theor. Appl. Genet..

[22] Giammaria V., Grandellis C., Bachmann S., Gargantini P.R., Feingold S.E., Bryan G., Ulloa R.M. (2011). StCDPK2 expression and activity reveal a highly responsive potato calcium-dependent protein kinase involved in light signalling. Planta.

[23] Zou J.J., Wei F.J., Wang C., Wu J.J., Ratnasekera D., Liu W.X., Wu W.H. (2010). *Arabidopsis* calcium-dependent protein kinase CPK10 functions in abscisic acid and Ca^2+^-mediated stomatal regulation in response to drought stress. Plant Physiol..

[24] Jing X., Tian Y.S., Peng R.H., Xiong A.S., Zhu B., Jin X.F., Gao F., Fu X.Y., Hou X.L., Yao Q.H. (2010). AtCPK6, a functionally redundant and positive regulator involved in salt/drought stress tolerance in *Arabidopsis*. Planta.

[25] Najar M.A., Rex D.A.B., Modi P.K., Agarwal N., Dagamajalu S., Karthikkeyan G., Vijayakumar M., Chatterjee A., Sankar U., Prasad T.S.K. (2021). A complete map of the Calcium/calmodulin-dependent protein kinase kinase 2 (CAMKK2) signaling pathway. J. Cell Commun. Signal..

[26] Harmon A.C., Gribskov M., Harper J.F. (2000). CDPKs-a kinase for every Ca^2+^ signal?. Trends Plant Sci..

[27] Pérez-García E.A., Meave J.A. (2005). Heterogeneity of xerophytic vegetation of limestone outcrops in a tropical deciduous forest region in southern México. Plant Ecol..

[28] Wang Q., Yu F.F., Xie Q. (2020). Balancing growth and adaptation to stress: Crosstalk between brassinosteroid and abscisic acid signaling. Plant Cell Environ..

[29] Wu P., Zhou H., Cui Y.C., Zhao W.J., Hou Y.J., Tan C.J., Yang G.N., Ding F. (2022). Stoichiometric characteristics of leaf, litter and soil during vegetation succession in Maolan National Nature Reserve, Guizhou, China. Sustainability.

[30] Atif R.M., Shahid L., Waqas M., Ali B., Rashic M.A.R., Azzem F., Nawaz M.A., Wani S.H., Chuang G. (2019). Insights on calcium-dependent protein kinases (CPKs) signaling for abiotic stress tolerance in plants. Int. J. Mol. Sci..

[31] Luo Y., Shi C., Yang S., Liu Y., Zhao S., Zhang C. (2023). Characteristics of soil calcium content distribution in karst dry-hot valley and its influencing factors. Water.

[32] Sanders D., Pelloux J., Brownlee C., Harper J.F. (2023). Calcium at the crossroads of signaling. Plant Cell..

[33] Ranty B., Aldon D., Cotelle V., Galaud J.P., Thuleau P., Mazars C. (2016). Calcium sensors as key hubs in plant responses to biotic and abiotic stresses. Front. Plant Sci..

[34] Wang C., Tang R.J., Kou S., Xu S., Lu Y., Rauscher K., Voelker A., Luan S. (2024). Mechanisms of calcium homeostasis orchestrate plant growth and immunity. Nature.

[35] Hirschi K.D. (2004). The calcium conundrum. Both versatile nutrient and specific signal. Plant Physiol..

[36] Kihana M., Yamagami M. (2022). Inhibitory effect of calcium on caesium absorption in plant roots. Radiat. Prot. Dosim..

[37] Negi N.P., Prakash G., Narwal P., Panwar R., Kumar D., Chaudhry B., Rustagi A. (2023). The calcium connection: Exploring the intricacies of calcium signaling in plant-microbe interactions. Front. Plant Sci..

[38] Tiffany Y.D., Marie B. (2019). Properties and functions of calcium-dependent protein kinases and their relatives in *Arabidopsis thaliana*. New Phytol..

[39] Weckwerth P., Ehlert B., Romeis T. (2015). *ZmCPK1*, a calcium-independent kinase member of the *Zea mays* CDPK gene family, functions as a negative regulator in cold stress signalling. Plant Cell Environ..

[40] Zhang Y.J., Bai D.F., Muhammad A., Li Z., Fang J.B., Zhong Y.P. (2021). Identification of CDPK family genes and their response to abiotic stress in Actinidia valvata. J. Fruit Sci..

[41] Zhao Y.L., Du H.W., Wang Y.K., Wang H.L., Yang S.Y., Li C.H., Chen N., Yang H., Zhang Y.H., Zhu Y.L. (2021). The calcium-dependent protein kinase *ZmCDPK7* functions in heat-stress tolerance in maize. J. Integr. Plant Biol..

[42] Gong L.S., Xiang Z.X., Wang Z., Lu M., An H.M. (2023). Identification of *CDPK* family genes in Cili (*Rosa roxburghii* Tratt.) and its expression in response to calcium levels. J. Fruit Sci..

[43] Zhao J., Zhu K.K., Fan P.H., Tan P.P., Peng F.R., Li Y.R. (2022). Identification and ex-pression analysis of CDPK gene family in pecan (*Carya illinoinensis*) and Chinese hickory (*Carya cathayensis*). J Agric. Biotechnol..

[44] Zhang K., Han Y.T., Zhao F.L., Hu Y., Gao Y.R., Ma Y.F., Zheng Y., Wang Y.J., Wen Y.Q. (2015). Genome-wide identification and expression analysis of the CDPK gene family in Grape, *Vitis* spp.. BMC Plant Biol..

[45] Myers C., Romanowsky S., Barron Y., Garg S., Azuse C., Curran A., Davis R., Hatton J., Harmon A., Harper J. (2009). Calcium dependent protein kinases regulate polarized tip growth in pollen tubes. Plant J..

[46] Yu W.W., Chen Q.F., Liao K., Zhou D.M., Yang Y.C., He M., Yu L.J., Guo D.Y., Xiao S., Xie R.H. (2024). The calcium-dependent protein kinase CPK16 regulates hypoxia-induced ROS production by phosphorylating the NADPH oxidase RBOHD in Arabidopsis. Plant Cell.

[47] Du H., Chen J.J., Zhan H.Y., Li S., Wang Y.S., Wang W., Hu X.L. (2023). The roles of CDPKs as a convergence point of different signaling pathways in maize adaptation to abiotic stress. Int. J. Mol. Sci..

[48] Yue J.Y., Jiao J.L., Wang W.W., Jie X.R., Wang H.Z. (2023). Silencing of the calcium-dependent protein kinase *TaCDPK27* improves wheat resistance to powdery mildew. BMC Plant Biol..

[49] Klimecka M., Muszyńska G. (2007). Structure and functions of plant calcium-dependent protein kinases. Acta Biochim. Pol..

[50] Shi S., Li S., Asim M., Mao J., Xu D., Ullah Z., Liu G., Wang Q., Liu H. (2018). The *Arabidopsis* calcium-dependent protein kinases (CDPKs) and their roles in plant growth regulation and abiotic stress responses. Int. J. Mol. Sci..

[51] Liu C., Huang Y., Wu F., Liu W.J., Ning Y.Q., Huang Z.R., Tang S.Q., Liang Y. (2021). Plant adaptability in karst regions. J. Plant Res..

[52] Meng W.P., Ran J.C., Dai Q.H., Tu N., Leng T.J., Ren Q.Q. (2023). Morphological and physiological adaptation characteristics of lithophytic bryophytes to karst high calcium environment. BMC Plant Biol..

[53] Xu C., Yang H., Huang C., Lan M., Zou Z., Zhang F., Zhang L. (2023). Interaction mechanism of Fe, Mg and Mn in karst soil-mango system. Land.

[54] Chen C., Wu Y., Li J., Wang X., Zeng Z., Xu J., Liu Y., Feng J., Chen H., He Y. (2023). TBtools-II: A “one for all, all for one” bioinformatics platform for biological big-data mining. Mol. Plant.

[55] Kumar S., Stecher G., Tamura K. (2016). MEGA7: Molecular evolutionary genetics analysis version 7.0 for bigger datasets. Mol. Biol. Evol..

[56] Waterhouse A., Bertoni M., Bienert S., Studer G., Tauriello G., Gumienny R., Heer F.T., Beer T.A.P., Rempfer C., Bordoli L. (2018). SWISS-MODEL: Homology modelling of protein structures and complexes. Nucleic Acids Res..

[57] Gouet P., Courcelle E., Stuart D.I., Métoz F. (1999). ESPript: Analysis of multiple sequence alignments in PostScript. Bioinformatics.

[58] Bailey T.L., Boden M., Buske F.A., Frith M., Grant C.E., Clementi L., Ren J., Li W.W., Noble W.S. (2009). MEME SUITE: Tools for motif discovery and searching. Nucleic Acids Res..

[59] Lescot M., Déhais P., Thijs G., Marchal K., Moreau Y., Peer Y.V., Rouzé P., Rombauts S. (2002). PlantCARE, a database of plant cis-acting regulatory elements and a portal to tools for in silico analysis of promoter sequences. Nucleic Acids Res..

[60] Schmittgen T.D., Livak K.J. (2008). Analyzing real-time PCR data by the comparative C-T method. Nat. Protoc..

