# Supplementary material for: Genome-Wide Identification and Characterization of the CDPK Family of Genes and Their Response to High-Calcium Stress in Yinshania henryi
Source: Genes (Basel). 2025 Jan 20;16(1):109. doi: 10.3390/genes16010109 (PMC11764810; doi:10.3390/genes16010109)
Supplement: Supplementary file 1 [file genes-16-00109-s001.zip › Supplementary Figure S2.pdf]

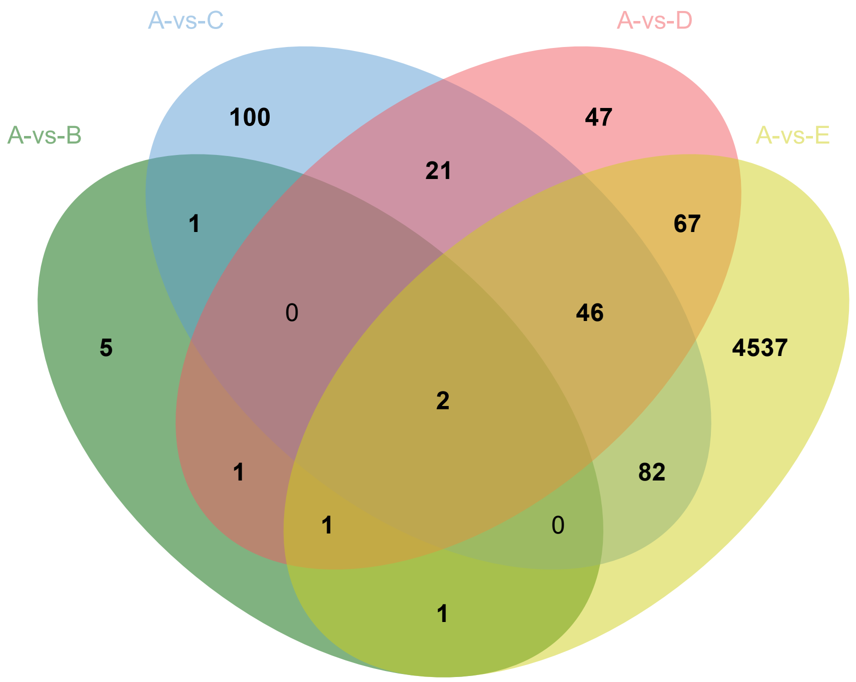

Figure S2: Comparison of differential expressed genes in different treatment groups. A, B, C, D, E represent CK, 10mM, 25mM, 50mM, and 100mM, respectively.
