# Supplementary material for: Genome-Wide Identification and Characterization of the CDPK Family of Genes and Their Response to High-Calcium Stress in Yinshania henryi
Source: Genes (Basel). 2025 Jan 20;16(1):109. doi: 10.3390/genes16010109 (PMC11764810; doi:10.3390/genes16010109)
Supplement: Supplementary file 1 [file genes-16-00109-s001.zip › Supplementary Figure S3.pdf]

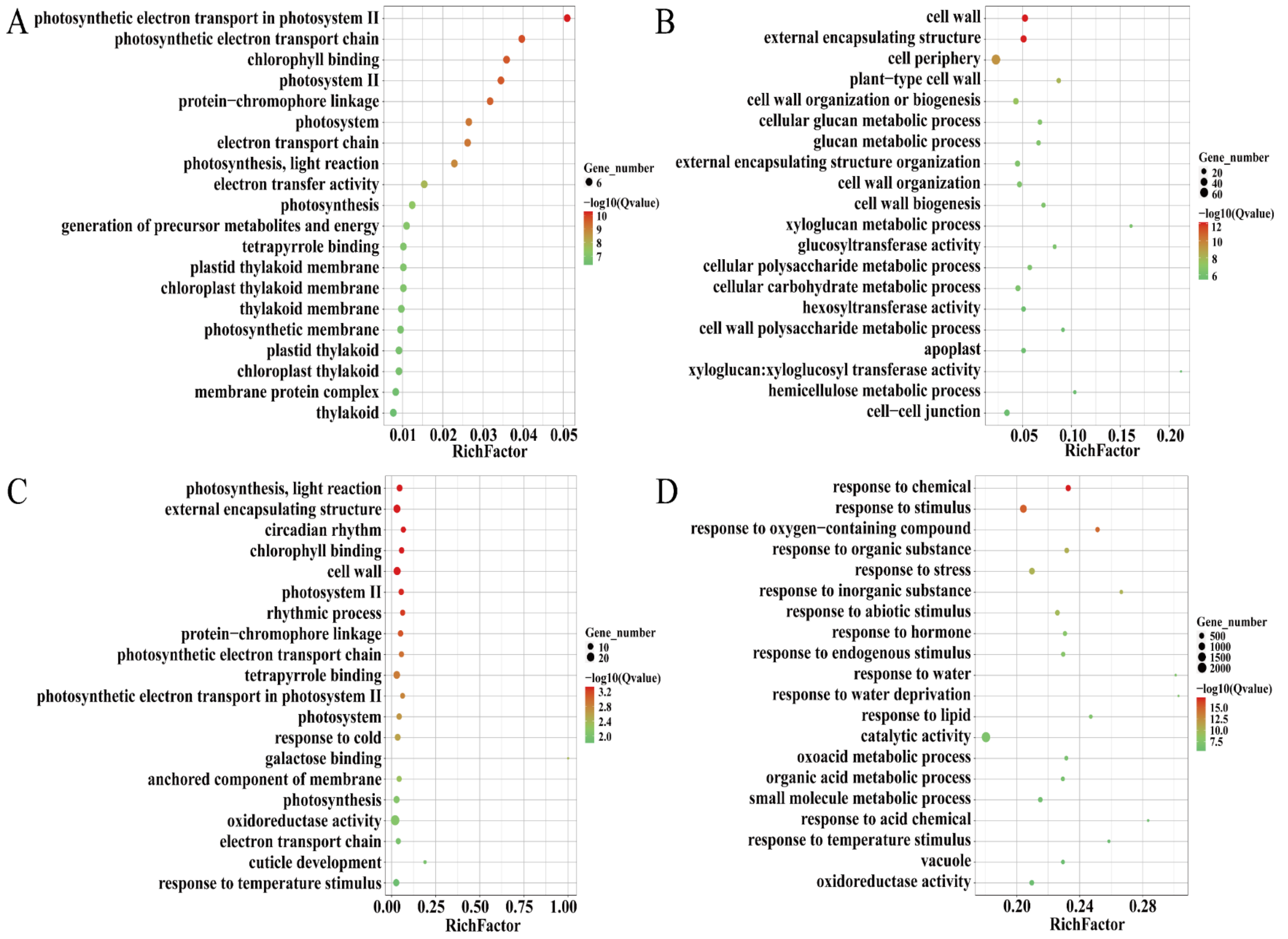

Figure S3: Significantly enriched GO terms of the DEGs generated in different groups. (A): CK-10 mM; (B): CK-25 mM; (C): CK-50mM; (D): CK-100 mM.
