# Supplementary material for: Genome-Wide Identification and Characterization of the CDPK Family of Genes and Their Response to High-Calcium Stress in Yinshania henryi
Source: Genes (Basel). 2025 Jan 20;16(1):109. doi: 10.3390/genes16010109 (PMC11764810; doi:10.3390/genes16010109)
Supplement: Supplementary file 1 [file genes-16-00109-s001.zip › Supplementary Figure S6.pdf]

A

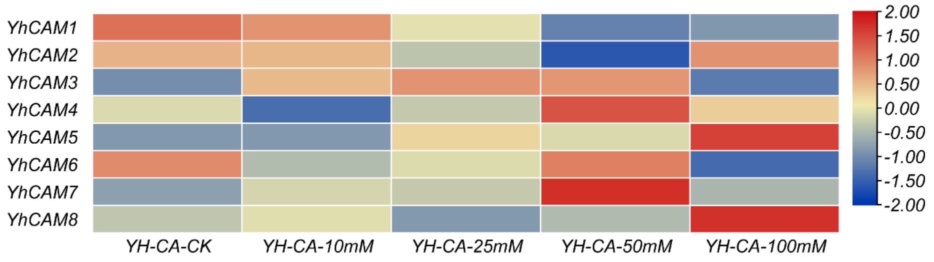

B

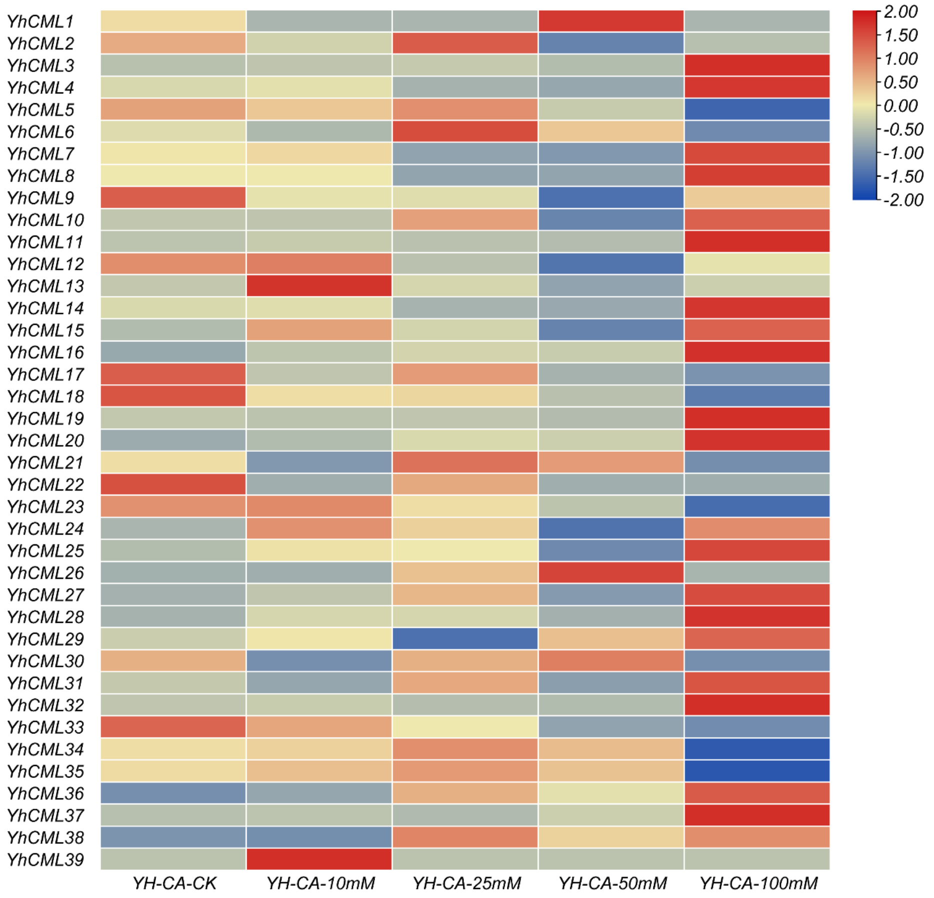

Supplementary Figure S6: Expression pattern of *YhCAM* genes (A) and *YhCML* genes (B) under $CaCl_2$ treatment of RNA-seq sequencing. RNA-seq data determined the relative expression levels. The CK (control), YH-Ca-10mM (10mM $CaCl_2$), YH-Ca-25mM (25mM $CaCl_2$), YH-Ca-50mM (50mM $CaCl_2$) and YH-Ca-100mM (100mM $CaCl_2$) represent different concentrations treatments. The transcript abundance level was normalized and hierarchically clustered using the log 2 (FPKM + 1) comparison among genes of different treatments. The expression value is presented on the color scale, with red representing high expression and blue representing low expression.
